# *In Vitro* Toxic Effects of Puff Adder (*Bitis arietans*) Venom, and Their Neutralization by Antivenom

**DOI:** 10.3390/toxins6051586

**Published:** 2014-05-19

**Authors:** Steven Fernandez, Wayne Hodgson, Janeyuth Chaisakul, Rachelle Kornhauser, Nicki Konstantakopoulos, Alexander Ian Smith, Sanjaya Kuruppu

**Affiliations:** 1 Department of Pharmacology, Monash University, Building 13E, Wellington Road, Clayton, Vic 3800, Australia; E-Mails: steven.fernandez@bakeridi.edu.au (S.F.); Wayne.Hodgson@monash.edu (W.H.); Rachelle.Kornhauser@monash.edu (R.K.); nickikonstas@hotmail.com (N.K.); 2 Department of Pharmacology, Phramongkutklao College of Medicine, Bangkok 10400, Thailand; E-Mail: jenjuth@yahoo.com; 3 Department of Biochemistry & Molecular Biology, Monash University, Building 77, Wellington Road, Clayton, Vic 3800, Australia; E-Mail: Ian.Smith@monash.edu

**Keywords:** antivenom, *Bitis arietans*, neurotoxicity, myotoxicity, cytotoxicity, procoagulant

## Abstract

This study investigated the *in vitro* toxic effects of *Bitis arietans* venom and the ability of antivenom produced by the South African Institute of Medical Research (SAIMR) to neutralize these effects. The venom (50 µg/mL) reduced nerve-mediated twitches of the chick biventer muscle to 19% ± 2% of initial magnitude (n = 4) within 2 h. This inhibitory effect of the venom was significantly attenuated by prior incubation of tissues with SAIMR antivenom (0.864 µg/µL; 67% ± 4%; *P* < 0.05; n = 3–5, unpaired *t*-test). Addition of antivenom at t_50_ failed to prevent further inhibition or reverse the inhibition of twitches and responses to agonists. The myotoxic action of the venom (50 µg/mL) was evidenced by a decrease in direct twitches (30% ± 6% of the initial twitch magnitude) and increase in baseline tension (by 0.7 ± 0.3 g within 3 h) of the chick biventer. Antivenom failed to block these effects. Antivenom however prevented the venom induced cytotoxic effects on L6 skeletal muscle cells. Venom induced a marginal but significant reduction in plasma clotting times at concentrations above 7.8 µg/100 µL of plasma, indicating poor procoagulant effects. In addition, the results of western immunoblotting indicate strong immunoreactivity with venom proteins, thus warranting further detailed studies on the neutralization of the effects of individual venom toxins by antivenom.

## 1. Introduction

*B. arietans* is a species of viper which occupies densely populated habitats throughout the Middle East and savannah areas of sub-Saharan Africa [1]. The precise number of human fatalities due to *B. arietans* envenoming is unknown. However, bites by this species are widely believed to contribute to a substantial proportion of the estimated 43,000 deaths from snake bite reported in Africa annually [2]. This makes *B. arietans* a significant public health concern in this region. The local effects of *B. arietans* envenoming include swelling, blistering, arterial thrombosis, bruising and necrosis [3]. The systemic effects of human envenoming include hypotension, bradycardia, spontaneous bleeding and thrombocytopenia [3]. Although death due to envenoming is rare, the absence of prompt treatment by antivenom can lead to poor quality of life as a result of disabilities due to local necrosis [4]. *B. arietans* venom is considered the most toxic of any viper species with a murine LD_50_ 9–13 µg/mouse. Polyvalent antivenom produced by South African Institute of Medical Research (SAIMR) is the treatment of choice following *B. arietans* envenoming [4]. This antivenom contains antibodies raised against a range of snake species (*B. arietans*, *B. gabonica*, *Haemachatus haemachatus*, *Naja melanoleuca*, *N. nivea*, *N. annulifera*, *Dendroaspis polylepis*, *D. jameson*, *D. angusticeps*, *N. mossambica*) found throughout Africa. 

Previous studies on *B. arietans* venom have resulted in the isolation of toxins including Bitanarin, a novel post-synaptic neurotoxin with PLA_2_ activity [5], bitiscetin, a platelet aggregation inducer [6], and Ba100, a toxin with fibrogenase activity [7]. In addition, the neutralisation of venom lethality by antivenoms raised in camels and horses has been examined [8]. Such studies however, fall short of determining the extent to which specific toxic effects are neutralized by the antivenom. Furthermore, no detailed studies have been conducted on the ability of SAIMR antivenom to neutralize the toxic effects of this venom.

Therefore, in this study, we examined the *in vitro* neurotoxic, myotoxic, procoagulant and cytotoxic effects of *B. arietans* venom, and the neutralisation of these effects with commercially available SAIMR antivenom.

## 2. Results and Discussion

### 2.1. Neurotoxicity

SAIMR antivenom is the treatment of choice following envenoming by *B. arietans* [4]. However, the manufacturers of SAIMR antivenom do not indicate the quantity of neutralising units in the antivenom. Previous studies from our laboratory show that SAIMR antivenom has a protein concentration of 180 mg/mL [9]. We therefore tested increasing concentrations of the antivenom in order to identify a minimum concentration which would prevent the toxic effects of the venom. Neurotoxicity is not a reported symptom of envenoming by *B. arietans*. However, the lack of neurotoxicity following envenoming does not rule out the presence of “weak neurotoxins” [10]. Similarly, the absence of neurotoxicity does not imply the lack of large presynaptic neurotoxins which may be found in low abundance, have limitations on binding to target receptors as well as distribution throughout the body. This has been the case with the potent presynaptic neurotoxin textilotoxin found in the venom of *Pseudonaja textilis* [11]. Despite the presence of textilotoxin neurotoxicity is not a symptom of envenoming by *P. textilis*, a phenomenon commonly referred to as “the brown snake paradox” [12]. Therefore we used the chick biventer cervicis nerve muscle preparation to examine *B. arietans* venom for the possible presence of neurotoxins.

**Figure 1:**
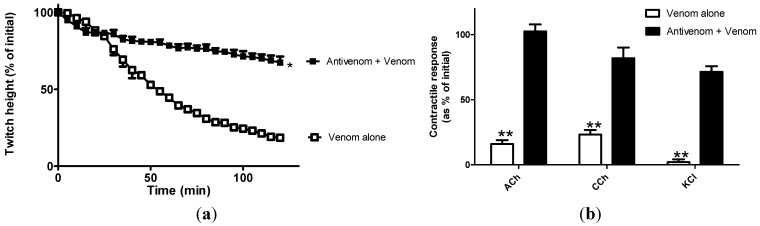
Neutralisation of *in vitro* neurotoxic effects by antivenom. Effect of venom (50µg/mL) alone and in the presence of South African Institute of Medical Research (SAIMR) polyvalent antivenom (0.864µg/µL) on the (**a**) nerve mediated twitches and (**b**) contractile responses to ACh, CCh or KCl in the chick biventer cervicis nerve muscle preparation. * Significantly different compared with *B. arietans* venom alone or ** venom + antivenom; *P* < 0.05; unpaired *t*-test; n = 3–5.

Venom (50 µg/mL; Figure 1a) produced a time dependent inhibition of nerve-mediated twitches in the chick biventer cervicis nerve-muscle preparation. Twitch height reduced by 50% (t_50_) within 53.0 ± 0.5 min. This can be classified as “weak” neurotoxicity given its manifestation at concentrations as high as 50 µg/mL, taking nearly 180 min to induce complete inhibition of nerve-mediated twitches. In contrast, previous work from our laboratory has indicated that death adder venoms at concentrations as low as 3 µg/mL can inhibit nerve mediated twitches of the chick biventer cervicis within 60 min [13,14]. Incubation of tissues with SAIMR antivenom (0.864 µg/µL) prior to the addition of venom significantly prolonged the time taken for complete twitch inhibition (>120 min). Lower concentrations of antivenom had no significant effect on the venom induced inhibition of nerve-mediated twitches of the CBCNM (data not shown).

**Figure 2:**
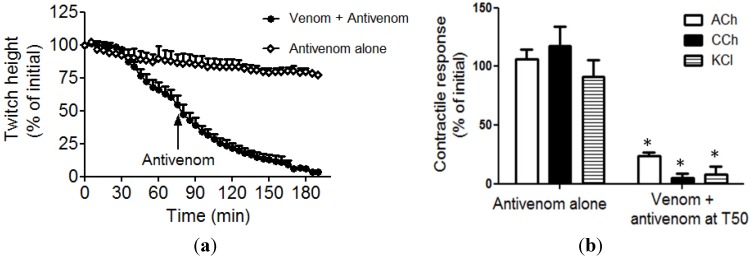
Reversal of neurotoxicity by antivenom. Effect of adding antivenom (0.864µg/µL) after the addition of venom (*i.e*., at t50 indicated by arrow) on (**a**) the nerve mediated twitches and (**b**) contractile responses to exogenous agonists of the chick biventer cervicis nerve muscle preparation n = 4; paired *t*-test; n = 4; * significantly different compared to antivenom alone.

The post synaptic nature of neurotoxicity was evidenced by the significant reduction in the response to exogenous ACh and CCh (Figure 1b). A unique post-synaptic neurotoxin, bitanarin, with phospholipase A_2_ (PLA_2_) activity has been isolated and characterised from *B. arietans* venom [5]. Bitanrin displaces (^125^I) iodinated α-bungarotoxin binding to nicotinic acetylcholine receptors from *Torpedo californica* with an IC_50_ of 4.3 ± 0.2 µM [5]. Bitanarin is found in low abundance in *B. arietans* venom (0.5% of dried whole venom), and thus may explain the observed weak neurotoxicity. The venom (50 µg/mL)-induced reduction in the response to exogenous agonists was prevented by prior incubation of tissues with antivenom (Figure 1b). The addition of antivenom (0.864 µg/µL) at t_50_ failed to reverse or prevent a further decrease in nerve-mediated twitches (Figure 2a) or responses to exogenous nicotinic agonists ACh and CCh (Figure 2b), of the chick biventer cervicis nerve-muscle preparation. KCl contracts the muscle directly, and the reduction in response to KCl is indicative of myotoxic damage to tissue, which is a frequently reported symptom of envenoming by this species [4,15].

### 2.2. Myotoxicity

Envenoming by *B. arietans* causes severe swelling and local necrosis [4]. While *in vivo* myotoxicity has been reported in mice, a polyvalent antivenom produced by the Instituto Butantan in Brazil has been shown to neutralise these effects [15]. However, the effect of SAIMR antivenom on the myotoxic effects of this venom is unknown. Given that SAIMR antivenom is the treatment of choice, it is important to assess the ability of this antivenom to neutralize the mytoxic effects. Therefore, using the chick biventer cervices muscle, we examined the ability of SAIMR antivenom to prevent *B. arietans* venom induced myotoxicity.

**Figure 3:**
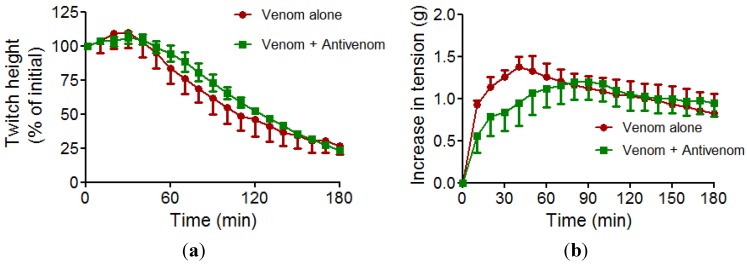
Myotoxic effects of *B. arietans* venom. The effect of venom (50 µg/mL) alone or in the presence of SAIMR antivenom (0.864 µg/mL) on (**a**) the direct twitches and (**b**) baseline tension of the chick biventer cervicis preparation (n = 4).

*In vitro* myotoxicity of venom (50 µg/mL) was evidenced by a time dependent decrease of twitches elicited by the direct stimulation of the CBCNM preparation (Figure 3a), while the baseline tension increased (Figure 3b). Prior incubation of tissue with antivenom (0.864 µg/µL) failed to prevent the decrease in twitch height or increase in tension.

This result further indicates that the lack of reversal of neurotoxicity by SAIMR antivenom is likely to be, at least partly, due to the myotoxic damage induced by the venom. However, the inability of SAIMR antivenom to prevent myotoxicity does not suggest the ineffectiveness of the antivenom. Although not practical in an organ bath environment, it is likely that increasing concentrations of antivenom beyond 0.864 µg/µL may prevent the observed myotoxic effects. This is further supported by the analysis of venom components by western blotting which indicate that antivenom binds venom toxins over a wide molecular mass range. The *in vitro* assay for myotoxicity used in this study cannot however distinguish between local or systemic myotoxic effects. Therefore, further detailed *in vivo* studies are required to determine if SAIMR antivenom can in fact prevent local as well as systemic myotoxic effects of *B. arietans* venom.

### 2.3. Effect of Venom on Cell Viability

**Figure 4:**
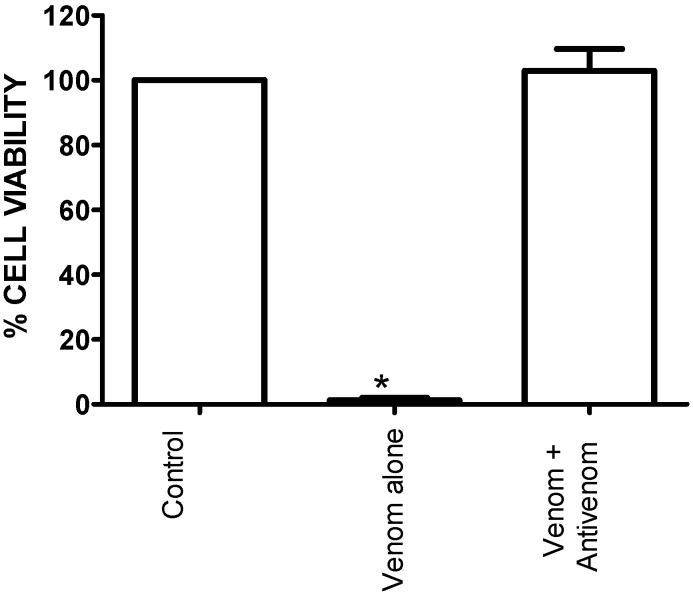
Neutralisation of *in vitro* cytotoxic effects by antivenom.Effect of venom (10 µg/mL) alone, or in the presence of antivenom (0.11 µg/µL) on the viability of L6 cells. * *P* < 0.05; significantly different compared with control (Cells + media), one-way ANOVA, n = 4.

Envenoming by *B. arietans* venom causes significant local swelling, blistering and necrosis [3]. Previous research indicates that these effects are the likely result of cytotoxins present in the venom. The presence of cytoxins has been reported in the venom of African spitting cobras, the envenoming by which causes painful swelling of the bite wound and local necrosis similar to that reported by *B. arietans* envenoming [16,17]. Therefore, using L6 skeletal muscle cells, we examined the *in vitro* cytotoxic effects of *B. arietans* venom and the ability of SAIMR antivenom to neutralize these effects. Treatment of L6 cells with *B. arietans* venom (10 μg/mL) caused a significant inhibition in cell viability. This was prevented by co-incubation with SAIMR antivenom at concentrations as low as 0.11 µg/µL (Figure 4).

### 2.4. 2D Gel Electrophoresis and Western Immunoblotting

The ability of antivenom to bind specific toxins in the venom is best studied via western immunoblotting. Therefore, in this study we subjected venom to 2D gel electrophoresis, followed by their transfer onto a PVDF membrane for detection by western blotting using SAIMR antivenom.

**Figure 5:**
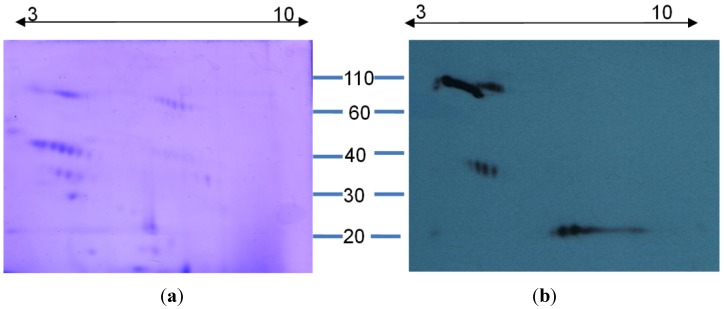
Analysis of venom proteins by 2D gel electrophoresis and western blotting. (**a**) *B. arietans* venom proteins (40 µg) were subjected to iso-electric focussing (pI indicated at the top) and resolved on 12% polyacrylamide gel; (**b**) Venom proteins resolved by 2D gel electrophoresis were detected by western blotting using SAIMR polyvalent antivenom (1:500 in 5% skim milk) and appropriate secondary antibodies. Molecular weight markers are indicated at the centre.

Coommassie staining of the 2D gel (Figure 5a) indicated the presence of venom proteins over the molecular mass range 5–110 KDa, with pIs over the range 3–8. Majority of the venom proteins appeared to be within the molecular mass range 30–50 KDa. The 2D gel also revealed the possible post-translational modification of several proteins as indicated by multiple spots for proteins of the same molecular mass. To the best of our knowledge this is the first report on the characterization of *B. arietans* venom by 2D gel electrophoresis.

SAIMR antivenom (0.360 µg/µL of total protein) appeared to only detect a few of the venom proteins visualized by coomasmsie staining (Figure 5b). Immunoreactivity was observed with acidic proteins over the molecular mass range 80–100 KDa and those at 35 KDa. Interestingly, antivenom detected toxins with an approximate molecular mass of 20 KDa and a corresponding pI over the range 5–8, which were not visible by coommassie staining. Our results warrant the purification of individual toxins, so as to facilitate in depth studies aimed at identifying those that can be neutralized by SAIMR antivenom.

### 2.5. Coagulation Studies

Haemostatic disturbances including spontaneous bleeding has been reported following *B. arietans* envenoming [3]. Therefore, in this study we have used a previously described *in vitro* turbidometric clotting assay to determine the effect of venom on blood coagulation [18]. Clotting time of plasma alone was 480 ± 10 s. Venom (7.8–500 µg/100 µL of plasma) induced a reduction in plasma clotting time (400 ± 10 s–370 ± 10 s respectively; significantly different compared to plasma alone, *P* < 0.05; n = 3; unpaired *t*-test). The effect of antivenom was determined using a submaximal venom dose of 250 µg/100 µL of plasma. Antivenom (18–54 µg/µL) failed to induce a significant delay in the plasma clotting time (250 ± 38.5 s–225 ± 41.3 s respectively; n = 6) compared to venom alone. Although *B. arietans* venom induces a significant delay in plasma clotting times, this effect is observed at much higher concentrations of venom than that reported for *T. carinatus*, *N. scutatus and H. stephensii*, where reduction in clotting times also occur at concentrations less than 10 ng/mL [19]. This is indicative of poor pro-coagulant effects in the venom of *B. arietans*. Our results warrant further detailed *in vitro and in vivo* studies aimed at delineating the mechanism(s) responsible for these hemostatic disturbances observed following *B. arietans* envenoming.

## 3. Experimental Section

### 3.1. Drugs and Chemicals

Bovine serum albumin (BSA), acetylcholine chloride (ACh), carbamylcholine chloride (CCh) and d-tubocurarine chloride were purchased from Sigma-Aldrich (St. Louis, Missouri, USA). Foetal Calf Serum (FCS) was purchased from CSL Ltd., Melbourne, Australia. BCA protein assay kit and CellTiter 96^®^ AQueous One Solution Cell Proliferation Assay (MTS assay) were purchased from Pierce biotechnology, Illinois, USA and Promega, Melbourne, Australia, respectively.

### 3.2. Venoms and Antivenoms

Freeze-dried *B. arietans* venom was purchased from Venom Supplies (Tanunda, South Australia). Venom stock solutions were prepared by reconstituting freeze-dried venom in MilliQ water when required. Reconstituted venom was kept at 4 °C and used within 8 h. SAIMR polyvalent antivenom was purchased from South African Vaccine Producers (SAVP) Ltd., Johannesburg, South Africa. Protein content of venoms and antivenoms were determined using a BCA protein assay kit (Biorad) according to manufacturer’s instructions.

### 3.3. Neurotoxicity Studies

Chickens (4–10 days old) were killed by CO_2_ inhalation followed by exsanguination, the biventer cervicis nerve-muscles removed and mounted under a resting tension of 1 g in 5 mL organ baths containing physiological salt solution of the following composition (in mmol/L): NaCl 118.4, NaHCO_3_ 25, glucose 11, KCl 4.7, MgSO_4_ 1.2, KH_2_PO_4_ 1.2 and CaCl_2_ 2.5. The solution was maintained at 34 °C and bubbled with carbogen (95% O_2_ and 5% CO_2_). Nerve-mediated twitches were evoked by stimulating the motor nerve (0.1 Hz, 0.2 ms) at supra-maximal voltage using a Grass S-88 stimulator. d-Tubocurarine (10 μmol/L) was added to the organ bath and the subsequent abolition of twitches confirmed that they were nerve-mediated (*i.e*., indirect). The tissues were washed thoroughly and, in the absence of nerve stimulation, contractile responses to acetylcholine (ACh; 1 mmol/L for 30 s), carbachol (CCh; 20 μmol/L for 60 s) and potassium chloride (KCl; 40 mmol/L for 30 s) were obtained. The preparations were allowed to equilibrate for approximately 20–30 min under electrical stimulation before the addition of venom. In all experiments, venom was left in contact with the tissue until the twitches were abolished or for a maximum period of 120 min. At the conclusion of the experiment, contractile responses to ACh, CCh and KCl were repeated. Where indicated tissues were incubated with SAIMR antivenom (0.18–0.864 µg/µL of total protein) 10 min prior to the addition of venom. The twitch height was then monitored over a time period of 1–2 h. For reversal studies, antivenom was added at the t_50_ time point (*i.e*., time at which the twitch height had decreased by 50%), and monitored for a further two hours or until twitches were abolished.

### 3.4. Myotoxicity Studies

Chick biventer muscles were dissected as described above for neurotoxicity studies. However, the electrodes were placed around the belly of the muscle and the preparation stimulated every 10 s with pulses of 2 ms duration at supramaximal voltage. d-tubocurarine (10 μmol/L) remained in the organ bath for the duration of the experiment in order to ensure specificity of muscle stimulation. Venom was left in contact with the muscle for 3 h or until twitches were abolished. Both twitch height and muscle tension were monitored throughout the course of the experiment. Where indicated tissues were incubated with SAIMR antivenom (0.18–0.864 µg/µL of total protein) 10 min prior to the addition of venom.

### 3.5. Cell Culture

L6 rat skeletal muscle cells were purchased from American Type Culture Collection (ATCC). These cells were cultivated and differentiated in 96 well cell culture plates as previously described [9].

### 3.6. Cell Proliferation Assay (MTS Assay)

#### 3.6.1. Effect of Venom

Media of L6 cells were removed. Venom stock solutions were diluted with DMEM containing 2% FCS and 1% penicillin/streptomycin (2% DMEM) to achieve concentrations between 10 and 150 μg/mL. These dilutions were added to wells in quadruplicate to the plate. Media controls (Cells + Media) and blanks were run in parallel. Cells were incubated at 37 °C with 5% CO_2_ for 24 h. After 24 h incubation, media was removed and wells were washed three times with pre-warmed PBS. 2% DMEM (50 μL) and MTS solution (10 μL) were added to the wells. Cells were then incubated at 37 °C in the presence of 5% CO_2_ for 3 h after which absorbance was measured at 492 nm using a Versamax plate reader.

#### 3.6.2. Antivenom Effects

2% DMEM was supplemented with venom or a mixture of venom and SAIMR polyvalent antivenom (0.11–0.53 µg/µL of total protein). Media controls, venom in media (without antivenom) and blanks were run in parallel. Cells were then incubated for 24 h at 37 °C in the presence of 5% CO_2_. After 24 h, wells were washed with pre-warmed PBS and cells were treated with MTS as described earlier (Section 3.6.1).

### 3.7. 2D Gel Electrophoresis and Western Immunoblotting

#### 3.7.1. Iso-Electric Focusing

Venom (40 μg) were applied to 7 cm immobilised linear pH gradient (pH 3–10) strips (IPG, Bio-Rad) with rehydration solution (Amersham, Uppsala, Sweden) and placed on a IEF cell system (Bio-Rad, Hercules, CA, USA) for iso-electric focusing. Electrical conditions were set as per manufacturer’s instructions starting with incubation at 50 V for 12 h.

#### 3.7.2. SDS-PAGE

After the first dimensional separation (Iso-electric focusing), the IpG strips were removed from the IEF system and first incubated with 5 mL of equilibration solution (50 nM Tris-HCl pH 8.8, 6M urea, 2% SDS, 30% glycerol, and traces of bromophenol blue) containing 0.05 g of DTT for 15 min followed by the incubation with 5 mL of equilibration solution containing 0.125 g of iodoacetamide for 15 min. Proteins were separated in the second dimension using 12% acrylamide gels. Protein spots were either visualised by coomassie brilliant blue staining or were subjected to western transfer and subsequent immune-detection.

#### 3.7.3. Western Immunoblotting

Venom proteins resolved on a 2D gel were then transferred onto a PVDF membrane which was then blocked with 5% skim milk. Membrane was then incubated SAIMR polyvalent antivenoms (1:500 diluted in 5% skim milk or 0.360 µg/µL) overnight. Immunoreactive bands were detected with appropriate secondary antibodies and ECL plus chemiluminescence reagent (Amersham Biosciences, Buckinghamshire, UK).

### 3.8. Coagulation Studies

#### 3.8.1. Effect of Venom

Pro-coagulant activity of venom was assessed using a turbidimetric assay described by O’Leary *et al*, [18]. Clotting times for each well were calculated as the time taken for kinetic absorbance to increase by more than 0.02 units compared to the average of the first two absorbance measurements. The venom was considered to have procoagulant activity if the clotting time was shorter than the plasma plus TBS (no venom) control clotting time. 

#### 3.8.2. Antivenom Effects

Where indicated the effect of antivenom (18–54 µg/µL) was tested against a final venom concentration of 250 µg/µL. TBS replaced the antivenom in “venom control” (*i.e*., TBS and venom only) and replaced both venom and antivenom in “plasma control” (*i.e*., TBS and plasma only) wells.

### 3.9. Statistical Analysis

Data are expressed as mean ± S.E.M. and were analysed using GraphPad Prism (GraphPad Software, San Diego, CA, USA). One-way ANOVA (analysis of variance) followed by Bonferroni’s multiple comparisons were used to analyse data from cell proliferation assay (MTS assay). Independent *t*-tests were used to analyse data from chick biventer studies. Differences were considered significant when *P* < 0.05.

## 4. Conclusions

In conclusion, we have for the first time examined the *in vitro* neurotoxic, myotoxic, cytotoxic and procoagulant effects of *B. arietans* venom, and the ability of SAIMR antivenom to neutralise these effects. Our *in vitro* studies indicate that SAIMR antivenom binds most of the venom proteins, and is effective in preventing the cytotoxic, neurotoxic and procoagulant effects of the venom. Antivenom however, failed to reverse the venom induced decrease in nerve-mediated twitches, which is the likely result of myotoxic damage. Antivenom (concentrations up to 0.864 µg/µL) also failed to prevent myotoxicity. Higher concentrations could not be tested in an organ bath environment without causing interference with the assay procedure. Our results warrant the isolation and subsequent characterization of the toxin(s) responsible for these effects, which would facilitate in depth studies on the efficacy of SAIMR antivenom in neutralizing these toxins.
